# High throughput method for detecting murine brain atrophy using a clinical 3T MRI

**DOI:** 10.1186/s12880-023-01124-0

**Published:** 2023-11-13

**Authors:** Michael Linzey, Krista DiSano, Nora Welsh, James C. Ford, Francesca Gilli, Heather Wishart, Andrew Pachner

**Affiliations:** 1 Integrative Neuroscience at Dartmouth, Dartmouth College, Hanover, NH US; 2 Department of Veterans Affairs Medical Center, White River Junction, Vermont, US; 3 Department of Psychiatry at Dartmouth Hitchcock Medical Center, New Hampshire, US; 4 Department of Neurology at Dartmouth Hitchcock Medical Center, Lebanon New Hampshire, US

**Keywords:** TMEV-IDD, Ventricular Volume, MRI, Brain Atrophy, Multi-animal imaging

## Abstract

**Background:**

There is a lack of understanding of the mechanisms by which the CNS is injured in multiple sclerosis (MS). Since Theiler’s murine encephalomyelitis virus (TMEV) infection in SJL/J mice is an established model of progressive disability in MS, and CNS atrophy correlates with progressive disability in MS, we used in vivo MRI to quantify total ventricular volume in TMEV infection. We then sought to identify immunological and virological biomarkers that correlated with increased ventricular size.

**Methods:**

Mice, both infected and control, were followed for 6 months. Cerebral ventricular volumes were determined by MRI, and disability was assessed by Rotarod. A range of immunological and virological measures was obtained using standard techniques.

**Results:**

Disability was present in infected mice with enlarged ventricles, while infected mice without enlarged ventricles had Rotarod performance similar to sham mice. Ventricular enlargement was detected as soon as 1 month after infection. None of the immunological and virological measures correlated with the development of ventricular enlargement.

**Conclusions:**

These results support TMEV infection with brain MRI monitoring as a useful model for exploring the biology of disability progression in MS, but they did not identify an immunological or virological correlate with ventricular enlargement.

**Supplementary Information:**

The online version contains supplementary material available at 10.1186/s12880-023-01124-0.

## Background

Multiple sclerosis (MS) is an inflammatory, demyelinating disease of the central nervous system (CNS) associated with disability progression in most patients. Although there are 23 FDA-approved therapies for MS which target inflammation, there are no medications that specifically address MS demyelination or disability progression, aspects of the disease which are poorly understood. Unlike lesional measures which do not correlate well with disability(“the clinico-radiologic paradox”) [1], CNS atrophy, measured by MRI, has had strong correlations with disability progression [2].

In order to learn more about CNS atrophy and progressive accrual of disability in MS, we utilized a mouse model of progressive neuroinflammation and neurodegeneration. Theiler’s murine encephalomyelitis virus-induced demyelinating disease (TMEV-IDD) is a virus-induced model that can recapitulate certain aspects of progressive MS, such as it is a chronic disease that features a slow accrual of disability. Cerebral atrophy in TMEV-IDD has recently been shown by one group to precede and predict disability progression [3, 4], which may serve as an important biomarker in this model. Further validation of this biomarker could assist in understanding the mechanisms of CNS damage in MS. We wished to confirm these findings and correlate cerebral atrophy, measured as ventricular enlargement by MRI, with immunological and neurobehavioral outcomes. To fully replicate the previously mentioned studies, we also focused our measurements on lateral ventricular enlargement because the lateral ventricles, in mice, contain most of the CSF space, by far. We have previously demonstrated that spinal cord MRI using diffusion tensor imaging (DTI) is very useful as a disability biomarker in TMEV-IDD [5]. However, ventricular volume assessment is easier, faster, and does not require a specialized animal MRI [6]. This technique would provide a more feasible option for many investigators working in MS animal models such as TMEV-IDD or experimental autoimmune encephalomyelitis (EAE).

## Methods

### TMEV-IDD induction

TMEV-IDD was induced by intracerebral injection of 4 × 10^6^ plaque-forming units of TMEV strain BeAn into 6- to 8-week-old SJL/J female mice, as previously described [7–10]. All experiments were approved by the Dartmouth Institutional Animal Care and Use Committee.

### Animals and imaging

Ten TMEV-infected mice and six uninfected (PBS- injected) sham controls were followed by MRI pre-infection-, and at 1-,2-,3-,4-, and 6-months post- infection. Mice were necropsied at month 6 p.i.(post-infection). T2 weighted sequences following a similar protocol to Herrmann et al. [11] (TSE, with TR = 2500, TE = 352, matrix = 256, and FOV = 84 in 144 ascending slices of 0.33 mm thickness, giving voxel resolution of 0.33 mm isotropic) were obtained (acquisition time = 19 min 56 s) using a Siemens Prisma (3 Tesla; Munich, Germany) horizontal bore clinical system in the Advanced Imaging Center at Dartmouth Hitchcock Medical Center. A custom manufactured system was designed to hold four mice adjacent to a commercially available single channel 70 mm animal coil (Philips 3T Integrated Optical Animal Solenoid RF-Coil, refitted with connectors for Siemens Prisma).

**Fig. 1:**
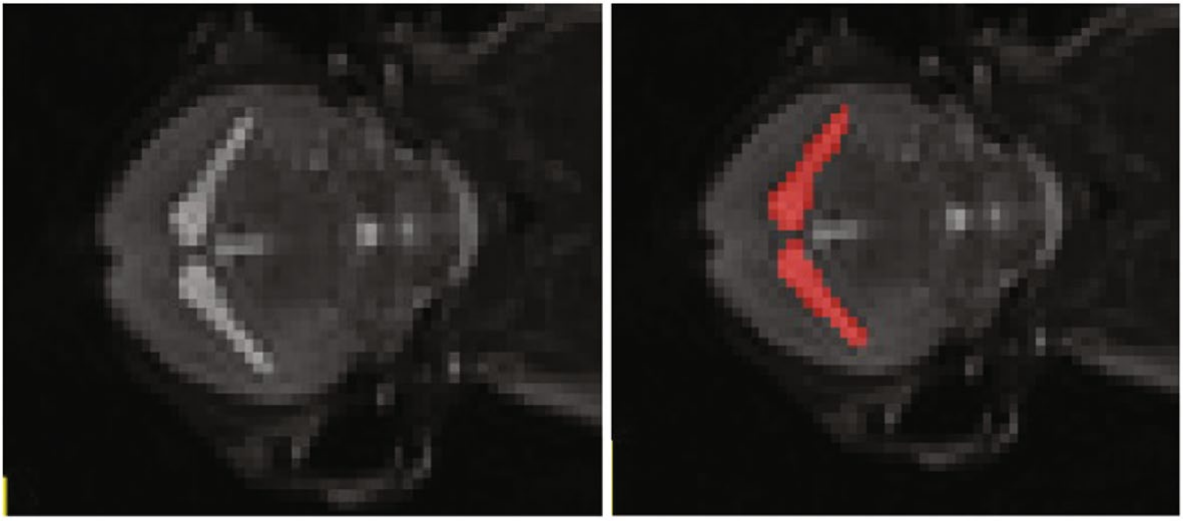
Representative axial brain MRI image from a TMEV-IDD mouse. Images before (left) and after (right) manual voxel segmentation highlighting lateral ventricles in red

The mice were in the supine position with their heads pointed towards the rear of the MRI. Inhalation anesthesia (1.5% isoflurane) was used for the imaging procedure. There was no loss of animals during the experiment, either to anesthesia or the natural course of the infection. An example of the type of image produced is shown in Fig. 1.

### ***Image ***analysis

Image analysis was performed using ITK-SNAP 3.8, from the Penn Image Computing and Science Laboratory*,* University of Pennsylvania, Philadelphia [12]. Prior to analysis, every image was preprocessed by one investigator (FG) to separate the four mice into separate image files. Each set of MRI scans were then independently analyzed by two different investigators (ML and KD) blinded to infection status. The analyzing investigators worked in collaboration with the institution’s Brain Imaging Lab to develop their standardized segmentation approach. Brightness and contrast were adjusted as needed to create well-defined CSF-parenchymal borders for each mouse. The lateral ventricular boundaries were then defined by manual segmentation of the region of interest (ROI) and ROI volume was calculated as the sum of the included voxels across all slices multiplied by the voxel volume.

The sum of left and right ventricular ROIs was used to determine a total ventricular volume (TVV) for each mouse. As expected, there was no significant difference in baseline TVV between the sham and infected mice (supplemental Fig. 1). However, because baseline TVV varied for each mouse, a delta TVV (ΔTVV) was calculated at each time point by subtracting the baseline TVV from the TVV at each time point. The ΔTTV was identified as enlarged above sham (E-TVV) if it was greater than the cutoff of the mean plus 2 standard deviations of the sham mice at the same time. The mouse was considered as being in the E-TVV group if it qualified as being E-TVV at any time point. A mouse was considered as being in the normal TVV group (N-TVV) if none of the 5 ΔTTV measurements was above the cutoff.

### Disability assessment, viral load, anti-TMEV antibody in CSF and serum, CNS IgG expression, and complement gene expression

These measures were obtained as previously described [13–15]. Progressive disability in mice was assessed by the Rotarod test, and the Rotarod data were expressed as a neurological function index (NFI) [13]. This is an index that uses a healthy baseline, unique for each mouse, and compares diseased run times to that baseline. Therefore, a score of 1 is indicative of no disability, while a score of 0.5, for example, indicates that the mouse could only remain on the rod for half the time at its baseline.

Real time-PCR (RT-PCR), using custom primers and probes for amplification of IgG1 and TMEV mRNA, were used to analyze gene expression in the spinal cord [14, 15]. Gene expression of the complement components C1q and C3, as well as glyceraldehyde phosphate dehydrogenase (GAPDH), were also determined in spinal cord tissue by real time RT-PCR. TaqMan Gene Expression Assays (ThermoFischer Scientific, Waltham, MA) were used as primers and probes for C1q, C3, and GAPDH. Transcript levels of IgG1, C1q, and C3 were expressed using relative quantification of the three target genes vs. GAPDH. TMEV mRNA levels were expressed as absolute expression against a plasmid standard curve.

Serum and cerebrospinal fluid (CSF) anti-TMEV binding antibody levels of individual mice were measured by a custom bead-based immunoassay with BeAN antigen.

### Statistical methods

Ventricular volume and gene expression data were assessed using a Pearson normality tests to determine significant deviations from a normal distribution. Based on the normality results, either the parametric Student’s t test or the non-parametric Mann–Whitney U test was utilized to compare groups. Linear regression analysis was used to determine a relationship between gene expression and clinical outcomes. All analyses were performed using Prism version 7.00 for Windows (GraphPad, San Diego CA), and all reported P values were based on two-tailed statistical tests, with a critical significant level of 0.05.

## Results

### Ventricular volumes

There were no significant differences detected between the left and right ventricles at any time point in either, sham or TMEV-IDD, group (Supplemental Fig. 2). Due to the lack of difference between the left and right ventricles, in all future measurements both ventricular volumes were added together to determine the total volume of CSF. Five TMEV-IDD mice had enlarged total ventricular volume (E- TVV), as defined above, and five TMEV-IDD mice had normal total ventricular volumes (N- TVV). The five E-TVV mice had 14/25 time point determinations above the cutoff (5 time points for 5 mice), and the five N-TVV mice, by definition, had 0/25 etermineations above the cutoff. Both sham and TMEV-IDD mice had increases in TVVs over time; by day 180 p.i., TTVs of sham mice increased by 43% on average, the N-TVV mice by 50%, and the E-TVV mice by 190%.

**Fig. 2:**
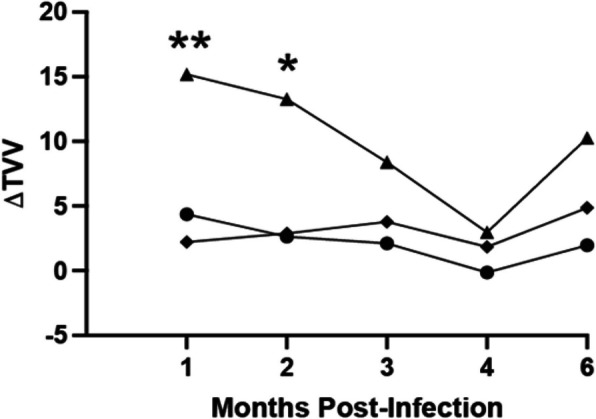
Delta total ventricular volume (ΔTTV) relative to baseline over time post infection (months). Median values shown for E-TTV mice (triangles), N-TTV mice (circles), and sham mice (diamonds). ΔTTV was significantly higher than sham at month 1 (*p* < 0.008) and month 2 (*p* < 0.027). Volume has been reported as mm^3^

Figure 2 shows the average ΔTVVs of the E-TVV, N-TVV, and sham mice at the different MRI time points. ΔTTV was significantly higher than sham at month 1 (*p* < 0.008) and month 2 (*p* < 0.027). There was a high degree of agreement between the investigators, with an intraclass correlation coefficient (ICC) of 0.983, indicating excellent reliability.

## Disability

**Fig. 3:**
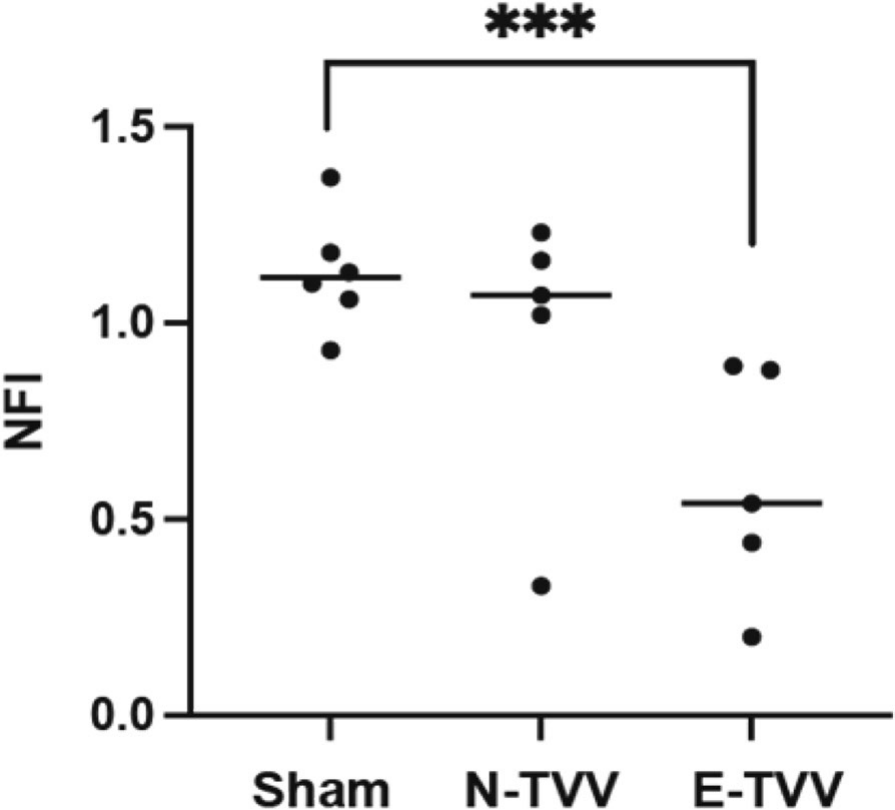
Rotarod performance disability scores of E-TTV mice, N-TTV, and sham mice. Scatter plots depict median NFI values for each group (bars) at day 180 p.i

As in previous TMEV-IDD experiments, sham mice slowly improved their Rotarod performance over time, while the Rotarod performance of the TMEV-IDD mice deteriorated over time. TMEV-IDD mice showed a range of diminished Rotarod performance, but as a group progressively worsened over time, with the median NFI of TMEV-IDD mice at 6 months p.i. being 62% that of the sham group. The five E-TVV mice had significantly lower NFI than the sham mice(*p* = 0.0032), while the N-TVV mice (Fig. 3) did not (*p* = 0.33).

### Immunological/virological measures

The data from all these measures was consistent with previous experiments on TMEV-IDD induced by the BeAn strain. All TMEV-IDD mice had strong anti-TMEV antibody responses intrathecally as manifested by high levels of anti -TMEV antibody in CSF and serum with all but one having an elevated index, consistent with intrathecal production of anti-TMEV antibody (Table 1). This data was reinforced by high levels of IgG1 gene expression in the brain and spinal cord (Table 1). In the five TMEV-IDD mice in which tissue was available to determine viral levels in the CNS, all had persistent virus at necropsy at 6 months p.i., sham mice were negative on all of these measures (Table 1). Gene expression for markers of an innate immune response in the CNS, C3 and C1q, were increased in TMEV-IDD mice, an average increase of threefold relative to sham (Table 1). Importantly, unlike Rotarod disability, there was no difference on any of these measures between the E-TVV and the N-TVV groups (Table 1).

**Table 1:** Immunological comparison of N-TVV and E-TVV mice. AU = arbitrary units; C1q = complement component C1q; and C3 = complement component 3

Immunological Outcomes	Sham (*N* = 6)	TMEV-IDD (*N* = 10)	*N*-TVV(*n* = 5) vs. E-TVV (*n* = 5)
**Number of Viral Copies**			
Brain (*n* = 5)	Negative	5.7E + 04	
Spinal Cord (*n* = 5)	Negative	9.9E + 06	
**Binding Antibody to Theiler's Virus**			
Serum (AU)	0	7.9E + 05 ± 2.2E + 05	*p* = 0.547
CSF (AU)	0	6.1E + 04 ± 5.9E + 04	*p* = 0.134
**IgG mRNA (2^-ΔCT)**			
Brain	1.8E-6 ± 1.1E-6	1.1E-3 ± 7.7E-4	*p* = 0.304
Spinal Cord	8.9E-4 ± 6.1E-4	2.3E-2 ± 3.2E-2	*p* = 0.315
**Complement Factors (2^-ΔCT)**			
C1q—Spinal Cord	6.6E-3 ± 2.4E-3	1.9E-2 ± 1.5E-2	*p* = 0.0966
C3—Spinal Cord	1.9E-2 ± 4.0E-3	3.1E-2 ± 1.5E-2	*p* = 0.837

## Discussion

The mechanisms of CNS destruction in MS leading to progressive neurological disability are unknown. Multiple beneficial and detrimental factors may be involved, among them inflammation, including both innate and adaptive immunity, neuronal injury, demyelination and remyelination, and plasticity. TMEV-IDD represents an excellent model of progressive disability associated with inflammatory demyelination. This allows teasing out of critical molecules involved in this complex process and identification of reliable CNS damage measures. In the experiments outlined above, we attempted to ascertain whether any of a range of immunological measures might correlate with enlarged ventricles, and to confirm previous findings that enlarged ventricular size in TMEV-IDD correlates with neurological disability [3].

Other groups have referred to their findings in rodent models as “brain atrophy”, and we agree that atrophy likely contributes to ventricular enlargement in this model. However, we choose to use the more precise term “enlarged total ventricular volume” (E-TVV) since we cannot rule out the possibility that other processes, e.g., hydrocephalus, may contribute to ventricular enlargement. In fact, given the considerable amount of CNS inflammation in TMEV-IDD [7, 16], hydrocephalus may be a contributing factor, especially considering the rapid development of E-TVV and its partial resolution over time, two features we observed that are less likely to occur if the cause was solely atrophy. This may indicate that TMEV-IDD may replicate disrupted CSF flow through the glymphatic pathways. A disruption to that flow could lead to the rapid ventricular enlargement and as that aspect of TMEV-IDD subsides the ventricles reduce in size but do not return to their baseline levels. This indicates some level of permanent damage and atrophy. Determining the precise cause(s) of E-TVV in this model will require larger MRI and pathology studies. This will provide the necessary data to determine the likely cause of the CNS injury that we observed.

Our data confirm that E-TVV does occur in the brains of TMEV-IDD mice, but this was a significant finding in only half of our mice. Our data also confirm that these E-TVV mice had worse neurological function as measured by Rotarod than those with normal total ventricular volumes(N-TVV). This indicates that E-TVV is a physiologically relevant biomarker for CNS injury in TMEV-IDD and those changes can be detected utilizing a clinical 3 T scanner. Another advantage of this MRI measure is that in 4 of 5 E-TVV mice, the TVV was already enlarged at one-month p.i., long before the development of disability, which usually begins around 75–90 days post infection. This finding raises the possibility that a clinical MRI could be used to identify mice that are more likely to develop weakness and are predisposed to the development and accumulation of disability.

There were some notable differences between our studies and results and those of the Mayo group [4]. First, we used the BeAn strain of TMEV while the Mayo group used the DA strain. There are apparent differences between the disease induced by these two strains [17], with BeAN-induced disease being generally less severe. However, it has not been shown that one strain is better or should be used preferentially over the other strain. Each strain, BeAN and DA, has its uses, but it is definitely possible that slight dfferences in viral strain activity could have caused the disparity in our results. Second, we used a 3 T clinical MRI scanner, which is designed for human imaging, while the Mayo group used a 7 T small bore animal machine, which afforded greater resolution. Third, the time course of ventricular enlargement in our mice peaked early, i.e., at one-month p.i. in our mice, while the peak in the Mayo studies occurred at 3 months p.i.

A major finding of this study is that our use of the 3 T clinical scanner demonstrates that this type of analysis of ventricular volumes can be performed without a specialized animal magnet of 7 T or higher field. The use of a 3 T clinical scanner is a real advantage given that many research centers will not have access to a high field small animal scanner. A further advantage of the 3 T scanner is that the larger bore of the 3 T scanner allowed 4 mice to be scanned simultaneously, which substantially reduced the time required per experiment because multiple mice can be tested simultaneously. However, given the lower resolution and sensitivity of the 3 T relative to the 7 T system, ventricular volumes determined by 3 T cannot be compared directly to those determined by 7 T systems and require comparison to internal controls, such as the sham mice used in our study. The precision of measurements is necessarily impacted by partial volume effects and a lower signal to noise ratio compared to scans at 7 T. It is possible that precision could be increased by more sophisticated surface-based modeling of the ventricles. A more advanced coil, use of contrast agents, or the addition of pulse sequences could also improve acquisitions sufficiently to increase data quality or reproducibility. However, the novelty and relevance of this study is that the scanning that was performed utilized widely available clinical sequences. This provides a potential solution for imaging centers that would like to perform preclinical MRI studies but do not have access to an animal MRI scanner.

A limitation of this study is that the ventricle volumes were manually calculated. This can be an issue due to possible bias or variability between the individuals calculating ventricular volume. We attempted to overcome this limitation by ensuring that those who manually calculated the volumes were blinded to the health status of the mice and to the volumes calculated by the other investigator. Using these strategies, we were still able to obtain a high level of rater similarity. However, as we continue this work, we expect that sophisticated automated segmentation approaches could be feasible in this domain and would leverage our initial manually segmented training data. Future efforts will also be made to perform larger studies with a cross-sectional design that will help reduce the baseline variance.

We chose measures primarily relevant to B cells, antibodies, and complement, for our immunological analyses, because B cell depleting drugs are highly effective in MS [18]. B cell active chemokines are correlated with MS neuroinflammation [19], and intrathecal antibodies [20, 21] have been implicated in CNS injury in the disease. None of these immunological measures correlated with the development of enlarged ventricular volumes. This is consistent with our previous results in TMEV-IDD, where multiple immunosuppressive or immunomodulatory medications have downregulated neuroinflammation without ameliorating disability progression [7–10]. It is also consistent with results in human MS, where most disability accrues in the secondary progressive phase of the disease. One of the most potent immunosuppressive MS drugs, natalizumab, did not reduce progression on the primary multicomponent disability endpoint used in a large, phase 3 randomized, double-blind, placebo-controlled trial in secondary progressive MS [22].

All these data indicate that we do not yet have an adequate understanding of the basic mechanisms of CNS injury in MS and its models, similar to our lack of understanding in other chronic neurological diseases such as Alzheimer’s disease, amyotrophic lateral sclerosis, and Parkinson’s disease. CNS atrophy occurs in numerous neurological disorders, and thus our finding has direct relevance to these other diseases. In addition, there is mounting evidence that there are immunological or viral elements to these other conditions as well. Therefore, understanding CNS atrophy in an immune-mediated, virally induced mouse model, like TMEV-IDD, could be useful to fields beyond MS. Magnetic resonance imaging in TMEV-IDD can thus provide a useful tool to address this formidable challenge facing the neuroscience community of identifying targets relevant to neurodegeneration in MS, and other conditions.

### Supplementary Information

**Additional file 1:** **Supplemental Figure 1.** Ventricular volume differences between the sham and TMEV-IDD MRI scans, at baseline. There was no statistical difference (*p*=0.367) in ventricular volumes between the sham and TMEV-IDD mice at the baseline scan. Volume has been reported as mm3 .

**Additional file 2:** **Supplemental Figure 2.** Ventricular volume differences between the right and left ventricles of sham and TMEV-IDD MRI scans. This was a longitudinal analysis that clearly shows that ventricular enlargement was not based on one side expanding more than the other. Volume has been reported as mm3.

## Data Availability

The datasets used and/or analyzed during the current study available from the corresponding author on reasonable request.
