# Supplementary material for: High throughput method for detecting murine brain atrophy using a clinical 3T MRI
Source: BMC Med Imaging. 2023 Nov 13;23:183. doi: 10.1186/s12880-023-01124-0 (PMC10641942; doi:10.1186/s12880-023-01124-0)
Supplement: Supplementary file 1 — Additional file 1: Supplemental Figure 1. Ventricular volume differences between the sham and TMEV-IDD MRI scans, at baseline. There was no statistical difference (p=0.367) in ventricular volumes between the sham and TMEV-IDD mice at the baseline scan. Volume has been reported as mm3 . [file 12880_2023_1124_MOESM1_ESM.pdf]

Supplemental Figure 1: A comparison of baseline ventricular volumes

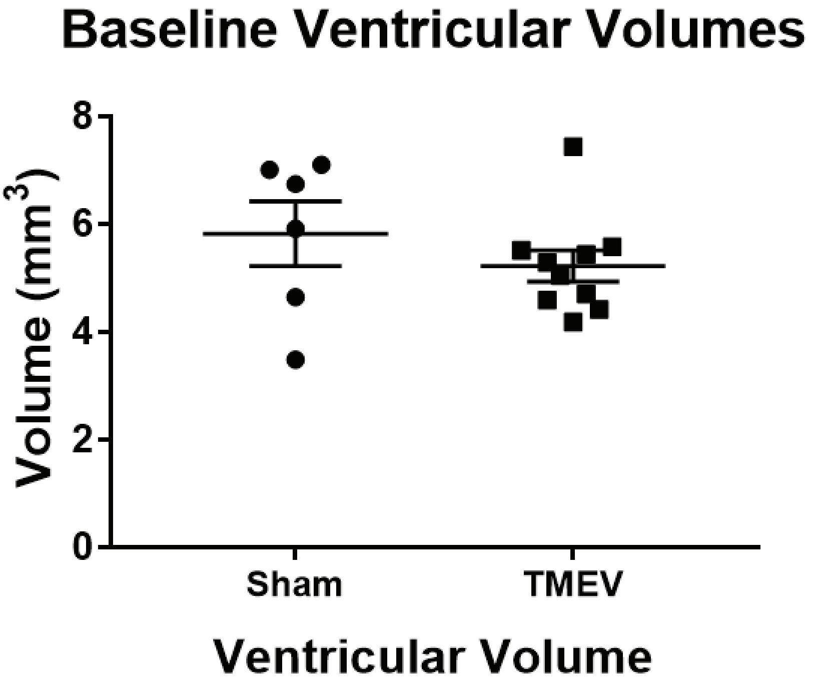

Supplemental Figure 1. Ventricular volume differences between the sham and TMEV-IDD MRI scans, at baseline. There was no statistical difference (p=0.367) in ventricular volumes between the sham and TMEV-IDD mice at the baseline scan. Volume has been reported as mm^3.
