# Supplementary material for: High throughput method for detecting murine brain atrophy using a clinical 3T MRI
Source: BMC Med Imaging. 2023 Nov 13;23:183. doi: 10.1186/s12880-023-01124-0 (PMC10641942; doi:10.1186/s12880-023-01124-0)
Supplement: Supplementary file 2 — Additional file 2: Supplemental Figure 2. Ventricular volume differences between the right and left ventricles of sham and TMEV-IDD MRI scans. This was a longitudinal analysis that clearly shows that ventricular enlargement was not based on one side expanding more than the other. Volume has been reported as mm3. [file 12880_2023_1124_MOESM2_ESM.pdf]

## Supplemental Figure 2: Comparing ventricular volumes between the right and left ventricles of sham and diseased mice

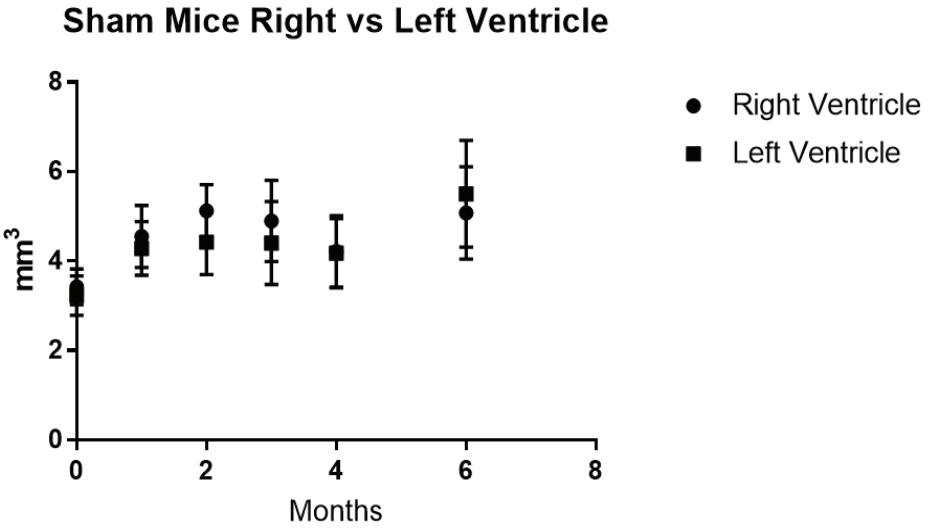


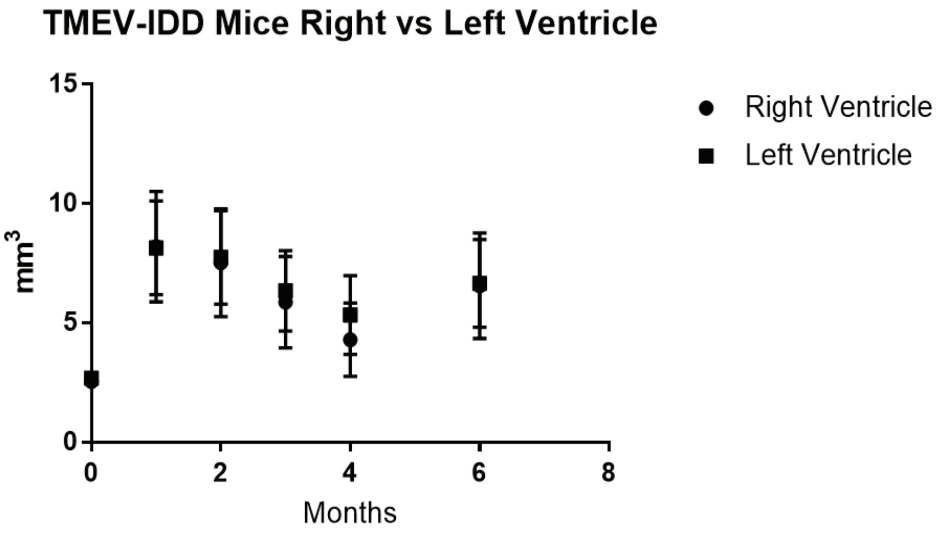


Supplemental Figure 2. Ventricular volume differences between the right and left ventricles of sham and TMEV-IDD MRI scans. This was a longitudinal analysis that clearly shows that ventricular enlargement was not based on one side expanding more than the other. Volume has been reported as $mm^3$.
